# Supplementary material for: Ligand discrimination in immune cells: Signal processing insights into immune dysfunction in ER+ breast cancer
Source: PLoS Comput Biol. 2025 Oct 30;21(10):e1013615. doi: 10.1371/journal.pcbi.1013615 (PMC12591468; doi:10.1371/journal.pcbi.1013615)
Supplement: Attachment — Submitted filename: Matache Response to Reviewers.docx [file pcbi.1013615.s003.docx]

**Response to reviewers**

**Reviewer #1:** This paper applies an information theoretic model to an experimental study of gene expression activation of immune cells under cytokine stimulation to understand immune dysfunction in ER+ breast cancer. The study is well motived given growing evidence for the central role of immune surveillance and its failure in cancer progression. The work provides a decent summary of relevant literature. There is prior work on information theoretic approaches to characterizing biological signaling networks of various kinds, but the approach here is still innovative and well differentiated from the prior work. The paper follows a reasonable strategy in modeling signaling in terms of a message (cytokine) that ultimately induce an output (pattern of gene expression activation) and seeking to understand how ability to correctly process that message is affected in cancers. The work focuses on a very specific model system, JAK/STAT signaling in ER+ breast cancers, and while it would be interesting to see the topic explored more broadly, the focus on a model system is understandably necessary given the experimental work involved. There are some important points of the approach that could be clearer, as explained below, but if we accept the model then the work leads to some interesting conclusions of immune dysfunction in cancers relative to healthy controls and of induction of cancer-like dysfunction through JAK/STAT inhibition. The results raise some questions of interpretation, however, also discussed in more detail below. Overall, it seems an interesting approach to an important topic although in need of at least more clarity and consideration of interpretation on some key points.

*We thank the reviewer for the careful consideration of the work. We have addressed the reviewer’s concerns in a point-by-point fashion below, noting that we do not have the ability to perform additional experiments. We note that the manuscript submission system applies compression to the figures. In order to view the figures clearly, please download the files with the link in the top right corner of the pdf.*

Major Criticisms:

R1.1. My largest question flows from some confusion about exactly how the computational analysis is done. The model is explained at a high level and the use of information theoretic concepts of signal to noise ratio (SNR) and error rate to quantify immune function seem sensible. But I did not follow where the canonical “correct” responses of each cell type to each stimulus are defined and are how they are applied. The model seems to imply that there is a correct output one expects to see with each stimulation, encoded as a set of ideal gene expression levels, but I did not see where these ground truth correct answers come from. Could the authors clarify?

*We thank the reviewer for noting this important aspect of the method, which we have now clarified in the introduction to the communication model section. Briefly, we consider the mean value of each transcription factor to be the ‘true’ signal, with the distribution around the signal to be the ‘noise’. A ‘correct’ response is then uniquely defined for each sample, which is an advantage of our approach, in that we consider the signal and noise characteristics from each individual patient/sample response, which may vary across individuals. We acknowledge the limitation of this approach in the discussion section.*

R1.2. A more focused technical question concerns the interpretation of the output as right or wrong based on whether the maximum likelihood assignment is the correct one. If one assumes the entire system is stochastic, then that output might itself be interpreted as a noisy input to downstream effects on gene regulation. If so, rather than saying that a stimulus produces one maximum likelihood output it might be more correct to say that a stimulus produces a probability density over possible outputs or a mixture of possible outputs. Subsequent analyses of SNR, error rate, and other information theoretic properties might be computed from this alternate interpretation much as they would be from the maximum likelihood one. I am not suggesting the authors need to redo the analysis with this alternative interpretation but would ask them to consider it and whether it might affect the conclusions.

*This is an excellent point that goes to the heart of many cell signaling analyses, that is, whether transcription factor phosphorylation is a reasonable proxy for cell signal processing, and to what degree the cell modulates the signal based on microenvironmental or other cell extrinsic cues before the cell performs a function in response. In the context of our analysis, an increased SNR could be a signature of stochasticity in output. Although not equivalent to the reviewer’s suggestion of a probability density function explanation, it is consistent and therefore would not affect the overall observations or conclusions.*

R1.3. My next concern relates to the previous ones but is also more of a question of interpretation: should we expect there to be a canonical correct response from each cell type under each kind of stimulation or would we expect that to be more condition specific? If I understand the paper correctly, it is defining responses in cancers as errors or noise because they are not the responses one sees in healthy controls. But perhaps they are the “correct” responses for a different context, e.g., someone experiencing high systemic inflammation. Are responses in cancers noisier than those in healthy controls or are they the correct responses for a different context? Information theory should have tools to distinguish between those possibilities, in asking whether there is greater entropy of responses in cancer versus healthy or comparable entropy around a different mean. Perhaps that is what is done here, but if so, I just was not clear on that.

*An important clarification of our approach is that there is no ‘biologically correct’ response, per say. There is only a response defined by the mean signal and noise defined by the variance of the multivariate distribution. The error rate and SNR derived from these definitions characterize the signal, which we compare between BC and healthy donors, and observe that BC samples display higher error rates and lower SNR. The concept of a ‘biologically correct’ response is a somewhat existential question that perhaps cannot be answered, as it is context dependent and likely defined by functional readouts rather than phosphorylation events. Our goal is to characterize signal propagation and fidelity using tools of information and communication theory. We have clarified this in the communication model and discussion sections of the manuscript.*

R1.4. To broaden the prior questions, I felt there is an unexplored issue of causality here that could use further consideration and perhaps some additional exploration. If we accept that cancer patients, and cancer patients with bad outcomes, have more immune dysfunction than healthy controls, what is the causality? Does immune dysfunction lead to more diagnosable cancer and worse outcomes due to failures of surveillance? Are aggressive cancers evolving to disrupt the immune system in ways that lead to worse outcomes? Are patients who experience bad outcomes developing comorbidities that lead to more immune dysfunction? Are patients who are fighting aggressive cancers exhausting their immune systems in ways that lead to dysfunction? All of the above? The authors touch on some of this in the Discussion but I would like to see a more thorough consideration of biological significance of the results and alternative hypotheses. I would not expect the authors to be able to rule out all of these ideas experimentally, but I would be interested in knowing more about what they think is happening and why and whether their data can support or rule out any particular interpretations. If not, it would be interesting to see a bit more discussion about hypothetical future work by which one might get at the causality more effectively.

*We thank the reviewer for challenging us to consider explanations for our observations. We have included the following as a new paragraph in the discussion section.*

*Although causality cannot be inferred from our data, we reason that signaling alterations observed in ER+ BC patients compared to healthy controls may be explained by inflammatory and cellular stress mechanisms. Elevated blood levels of IL-6 and IL-10 in ER+ BC suggest a pro-inflammatory environment that can chronically activate pathways such as IL-6/STAT3, which are known to correlate with poorer survival outcomes and may reflect more aggressive disease states(2). Prolonged exposure to elevated IL-6 and other cytokines circulating in the peripheral blood can induce changes in cell membranes, potentially affecting signal transduction without altering receptor expression(35). This immune stress and persistent inflammation may contribute to increased cellular entropy and systemic immune dysregulation, which are known hallmarks of cancer. Together, these factors may create a milieu in which signaling pathways are disrupted, even in the absence of changes in receptor levels, highlighting the impact of cytokine-driven stress and tumor-induced disruption and entropy in cellular communication, resulting in changes to SNR and increased signal detection error rates.*

R1.5. I also question whether some bias might be introduced by isolating PBMCs and effectively removing them from the natural context before conducting stimulation. Is there reason to be confident the response of isolated cells in culture would match that of the cells in situ? Even if not, it is still interesting that isolated cells from cancer patients behave differently from isolated cells from healthy controls, but perhaps it opens up other hypotheses for why there is a difference.

*Although ex vivo stimulation is not an approximation for in vivo signaling, the fact that we observe orders of magnitude differences in error rates and SNR between healthy donors and breast cancer samples indicates cell intrinsic mechanisms of signaling alterations in BC. We have added this consideration to the limitations paragraph of the discussion.*

Minor critiques

R1.6. I was confused by the text in the Discussion “This leads us to hypothesize that BC patients … in ways beyond simple suppression.” Could the authors elaborate on what was meant here?

*We have clarified the statement to read as follows:*

*In contrast, signaling kinase inhibiting therapies such as clinically approved JAK inhibitors may induce immune system dysregulation through reduction of SNR and amplification of Pe, which could be unintended downstream consequences of JAK suppression (7). Specifically, we observed that JAK1/2 inhibition resulted in a pattern of immune signaling dysregulation different from controls or BC samples, with statistically significant increases in error rates and decreases in SNR observed in all cell types as compared to BC samples, with the notable exception of the error rates in CD4+ T cell subsets as relative to controls (Figure 5D, S16).*

R1.7. Several of the figures use a particular plot style to show variability in error rates and SNRs (Figs 3A, 4A, 5A, 5B, S2, S5). The information conveyed by these plots is important but I have trouble distinguishing the colors of the spots without zooming in on an electronic copy to very high resolution, given the light coloring and the dark grid lines. I wonder if the authors might think of some way to make these plots larger and/or easier to see.

*We thank the reviewer for prompting us to improve the clarity of the results. We have reduced the number of gridlines and increased the marker size on some of the plots to improve clarity. Because these plots should be compared to each other, we chose to keep the y axis limits consistent. We have tightened the x axis (SNR) on all plots. We hope these revised figures are sufficiently legible.*

R1.8. In Figure 5C/D, I am not sure I quite buy that the ruxolitinib-treated healthy samples look like cancer samples. The three conditions each look quite distinct, which is not surprising but I feel is not the message the authors are conveying. I think it would be useful to be a bit more precise about exactly what is claimed in interpreting these figures and how it can be established with statistical significance.

*We agree with the reviewer that JAK inhibition results in a different signaling profile than either control or BC samples. We have revised the results and discussion accordingly. Statistical significance for SNR and Pe comparisons of ruxolinitib with BC samples is included now as a supplementary Figure S16. We found that adding *’s to indicate statistical significance to Figure 5C was too cluttered.*

R1.9. In Figure 5, the caption is missing a description for Fig 5D.

*We thank the reviewer for noting this omission. We have added a caption for Figure 5D.*

**Reviewer #2:** In this study, Matache, et al use an information theoretical approach to explore signal processing of immune cells captured in blood samples of a cohort of ER+ breast cancer patients and compare this to healthy controls. Consideration of how extracellular signals are encoded into responses, and how cells can discriminate between these signals is a unique approach not typically used. The analyses are comprehensive, but some of the biological rationale and interpretation seem overstated. Major and minor comments are outlined below.

Major comments

R2.1 Raw data: The study would benefit from including an overview of the data. For example: cell type population frequency; ligand-induced signal change as compared to untreated.

*We have included a new Table 1 that summarizes the data and experimental conditions in addition to the clinical variables provided in the supplement. Due to the large number of patients, samples, and stimulations, a table summarizing differences in any one feature, pSTAT3 for example, would not be helpful for the reader.*

R2.2 Confusion analysis: Many of the values in the confusion matrix are quite similar. The authors should comment on how biologically meaningful small differences are, and whether the ranks are over-interpreted. Based on prior literature about expected downstream signaling and impact of these cytokines, are the most commonly confused ligands expected?

*We thank the reviewer for noting this subtle point. Indeed, we relegated the confusion matrix plots to the supplement because the results for most cell types and ligand combinations were not insightful, with the exception of IL-2/IL-4 in CD4+ T cells, which we discuss in the main text.*

R2.3 JAK/STAT inhibition studies: Authors use the jak/stat inhibitor ruxolitinib “to investigate the clinical relevance of ligand identification error rates and signal fidelity”. The rationale for these studies is unclear. No malignant cells are being treated in this experiment, so the link to clinical relevance seems tenuous.

*We thank the reviewer for noting a conceptual gap in explaining our rationale for the ruxolitinib experiments. Because ruxolintinib is administered intravenously, its effects are experienced in the peripheral blood as well as in the tumor, moreover, in primary (not metastatic) breast cancer, there should be no (or few) malignant cells in the peripheral blood. We conducted an experiment on healthy donor cells to ask the question: how would JAK inhibition alter an otherwise healthy immune signaling profile, as we do not have access to peripheral blood of BC patients treated with systemic ruxolitinib. We have clarified this in the methods section.*

R2.4 The interpretation of decreased signal fidelity is also lacking. One explanation for decreased signal fidelity in the presence of the inhibitor is that the inhibitor induced a strong pathway inhibition that cannot be overcome by ligand stimulation. Better casting these findings in the context of prior literature of how microenvironmental signals can influence therapeutic response would be beneficial.

*We thank the reviewer for suggesting that we elaborate on our interpretation of the findings. Reviewer #1 also suggested more interpretation. We have added discussion including citations to literature in the discussion section.*

R2.5 Link to code base seems to be missing

*We apologize for the omission. Github and Zenodo links are now provided for both the computational codes and source data, respectively. Codes reproduce all reported results by simply executing two files: one for healthy donor samples and one for breast cancer samples.*

[*https://github.com/rrockne/BreastCancerSignalProcessing*](https://github.com/rrockne/BreastCancerSignalProcessing)

[*https://doi.org/10.5281/zenodo.16906422*](https://doi.org/10.5281/zenodo.16906422)

R2.6 Supplementary table of gated data are missing.

*All gated data are now provided on Zenodo. Gating strategy is shown in Figures S1 and S2.*

Minor comments

R2.7 Figure S8 – figure legend does not seem to reflect the data presented

*We have corrected the figure legend and thank the reviewer for catching this detail.*

R2.8 Figures S12-15 are only mentioned in the discussion. These should be mentioned in the main text

*As Figures S12-15 are effectively negative results, revealing no consistent correlation between receptor expression and our measures of signaling. As such, we did not see value in dedicating a section to the analysis or results in the main text. Rather, we prefer to summarize the findings in context of the discussion and provide all data and findings in the supplement. We hope the reviewer is satisfied with this approach.*
